# Fatiga de compasión en la atención primaria rural: un desafío silencioso para el cuidado profesional

**DOI:** 10.1016/j.aprim.2026.103445

**Published:** 2026-04-08

**Authors:** Edwin Gustavo Estrada-Araoz

**Affiliations:** Universidad Nacional Amazónica de Madre de Dios, Puerto Maldonado, Perú

*Sr. Editor,*

En la actualidad, para muchas personas que viven en zonas rurales, la atención primaria representa no solo el primer contacto con el sistema de salud, sino también el vínculo más cercano con la atención pública. En este contexto, el profesional cumple varios roles: médico, educador, acompañante y, muchas veces, sostén emocional de comunidades con recursos limitados y una alta demanda de atención. La exposición constante al sufrimiento y la identificación empática con los pacientes, sumadas a la carga asistencial y al aislamiento profesional, generan condiciones propicias para la fatiga de compasión^1^. Este fenómeno, entendido como una forma particular de desgaste emocional provocada por el contacto prolongado con el dolor ajeno, se ha convertido en un desafío silencioso para la continuidad y la salud del trabajo en el primer nivel de atención.

A diferencia del síndrome de *burnout*, centrado en la sobrecarga laboral, la fatiga de compasión está relacionada con la respuesta empática que el profesional mantiene frente al sufrimiento ajeno. En las zonas rurales, donde los límites entre la vida personal y la práctica sanitaria se confunden, la carga emocional se hace más intensa. Atender a pacientes que forman parte del propio entorno, enfrentar la muerte sin apoyo especializado o convivir con carencias estructurales genera sentimientos de impotencia, distanciamiento o culpa. Se ha reportado que este tipo de fatiga que afecta al personal de atención puede ocasionar una menor empatía, menor satisfacción laboral y una mayor intención de abandonar la práctica clínica^2^.

A pesar de su impacto, este tipo de desgaste emocional sigue sin recibir la atención que merece en las políticas de salud pública. Los programas de bienestar laboral suelen centrarse en aspectos administrativos o en el estrés asociado a la carga asistencial, sin considerar el costo afectivo del trabajo empático. La evidencia demuestra que las intervenciones que fortalecen la resiliencia y la regulación emocional pueden reducir la fatiga de compasión y mejorar la sensación de eficacia personal^3^. En las zonas rurales, esto cobra aún más sentido, porque el acceso a apoyo psicológico y a redes de supervisión profesional es escaso.

La gestión de los recursos humanos en el primer nivel de atención debe incluir también la dimensión emocional del trabajo sanitario. Las reuniones de equipo, las tutorías entre pares y los espacios de reflexión sobre la práctica clínica representan alternativas viables y de bajo costo que funcionan como sostén colectivo. Asimismo, incorporar contenidos de autocuidado y afrontamiento compasivo en los programas de capacitación continua favorece la estabilidad emocional del personal y mejora la calidad del servicio que se ofrece.

En conclusión, la fatiga de compasión no debe verse como una debilidad personal, sino como el resultado de trabajar en entornos donde las emociones se viven con intensidad. Reconocerla y actuar para prevenirla es parte del compromiso que las instituciones deben asumir con quienes sostienen el primer nivel de atención. Cuando se crean condiciones laborales más humanas y sostenibles, se fortalece la motivación, la empatía y la calidad del servicio. Proteger el bienestar del personal rural significa, en última instancia, proteger la salud de las comunidades que dependen de su dedicación.

## Financiación

La presente carta no ha recibido ningún tipo de financiación pública ni privada.

## Autoría

El manuscrito fue concebido, redactado y revisado en su totalidad por el autor, quien aprueba la versión final y asume plena responsabilidad por su contenido.

## Consentimiento informado

Dado que el contenido no incluye datos personales, historias clínicas ni testimonios de personas, no se requirió consentimiento informado.

## Consideraciones éticas

Esta carta al editor no presenta resultados de investigación ni información proveniente de participantes humanos o animales. Por tal motivo, no fue necesaria la aprobación por parte de un comité de ética en investigación.

## Conflicto de intereses

El autor declara que no existe ningún conflicto de intereses relacionado con el contenido de este manuscrito.
